# Schedule risk model of water intake tunnel construction considering mood factors and its application

**DOI:** 10.1038/s41598-024-54261-z

**Published:** 2024-02-15

**Authors:** Xin Li, Wei Sun, Honggang Fu, Qingsheng Bu, Zhiguang Zhang, Jian Huang, Dongnian Zang, Yuan Sun, Yong Ma, Rui Wang, Jingjing Hu, Yanan Shen

**Affiliations:** 1 Jiangsu Collaborative Innovation Center for Building Energy Saving and Construct Technology, Jiangsu Vocational Institute of Architectural Technology, Xuzhou, 221116 China; 2 Department of Building Intelligence, Jiangsu Vocational Institute of Architectural Technology, Xuzhou, 221116 China; 3 Jinggu Environment Construction Co., Ltd, Nanjing, 211000 China; 4 Sinohydro Engineering Bureau 7 Co., Ltd, Chengdu, 610081 China; 5 Xuzhou Water Conservancy Engineering Construction Supervision Center Co., Ltd, Xuzhou, 221000 China; 6 Xuzhou Hydraulic Engineering Construction Co., Ltd, Xuzhou, 221006 China; 7 Suyu Bureau of Water Resources in Suqian City, Suqian, 223800 China; 8 Jiangsu Water Conservancy Construction Engineering Co., Ltd, Yangzhou, 225100 China; 9 Xuzhou Water Conservancy Engineering Operation Management Center, Xuzhou, 221018 China; 10 Yishu River Water Conservancy Administration, Linyi, 276001 China

**Keywords:** Engineering, Civil engineering

## Abstract

The mood index $r$ was used to describe evaluator attitudes regarding the progress of a project that formed the basis of a construction period prediction model. The degrees of pessimism $\alpha$ and optimism $\beta$ were introduced, and an analysis model was established using $\alpha$ and $\beta$ to predict the construction period and completion probability Firstly, the absolute construction period of each process of tunnel No. 2 can be obtained according to the measured daily average footage of each process of tunnel No. 1. Secondly, the probability of the stoppage caused by different factors can be obtained after the statistical analysis of the factors responsible for the stoppage of tunnel No. 1. Finally, the expected construction period and completion probability of tunnel No. 2 under different pessimism and optimism conditions are obtained by using the progress risk analysis theory of emotional models and the program evaluation and review technique method. An engineering application showed that the expected construction period increased, and the completion probability decreased considerably with increasing pessimism; the opposite trend occurred as optimism increased. During the process of risk management and control, the prediction model can be used to perform precise quantitative analysis of the expected construction period and completion probability, reduce the blindness of construction management, control decisions of complex giant tunnel projects, and provide a more accurate basis for decision makers to judge risks. The findings of this study can be applied to hydraulic tunnels and can provide a reference for traffic tunnels, railway tunnels, and other similar projects.

## Introduction

Water intake tunnel construction is a systematic project planning phase involving several influencing factors that have complex relationships^1^. This type of construction represents a complex undertaking with multiple process combinations, lapping, and a limited working surface. Furthermore, the construction intensity is high and the mutual interference among different factors is considerable. The actual construction progress is dynamic and variable, and most projects deviate from the original plan, thus increasing the difficulty of controlling real-time construction progress^2^. Different researchers have investigated the use of methods for the prediction and control of the risks associated with construction progress. Zhang et al.^3^ comprehensively used the confidence index method and analytic hierarchy process (AHP) to analyze and evaluate the risks during tunnel construction. Zhuo et al.^4^ used the fuzzy comprehensive evaluation method to establish a construction risk evaluation model considering the risk probability and consequences. Zhou^5^ proposed a landslide-risk-based, early warning method based on the efficacy coefficient method and advanced geological predictions. Yu et al.^6^ proposed a probabilistic risk analysis approach for diversion tunnel construction simulations. Kim et al.^7^ generated and evaluated excavation schedules for use in the preconstruction and construction of the hard rock tunnels. Mahmoodzadeh et al.^8^ used artificial intelligence tools for decision-making in tunneling. Xu et al.^9^ constructed a dynamic Bayesian-based risk assessment model for shield tunnel construction from the perspective of changing construction stages and activities. Thapa et al.^10^ considered the most successful empirical risk-minimization-based Kriging model and the structural risk-minimization principle-based support vector regression model for comparison. Wu et al.^11^ performed construction safety risk assessments and generated early warnings of nearshore tunnels based on BIM Technology. Kwon et al.^12^ developed an advanced tunnel risk management model by combining the analytic hierarchy process (AHP) and fuzzy set theory.

Existing research on risks to underground construction schedules has primarily focused on the quantitative analyses of the risk factors using the AHP, fuzzy evaluation method^13^, neural network algorithms, extension theory, and other methods^14^. Researchers working in this field have also applied other approaches to study delay risks. Starting from the perspective of the system theory and using the principles of statistics and risk aggregation to plan judiciously the production time of a project^15^ proposed a new method to set the key chain buffer in the progress control of the interval tunnel construction. Xiao et al.^1^ studied the probability distribution of the risk factors using the correlated schedule risk analysis model method by considering the potential risks in the process of water intake during tunnel construction. Sharafat et al.^16^ analyzed the risk associated with tunnel boring machine (TBM) tunneling projects performed in difficult ground conditions based on the conventional bow-tie risk analysis approach.

In research related to the attitude of the evaluator and project risk^17^ explored the impact of risk attitude on the assessment of project uncertainty, encompassing risk and opportunity and expected project performance. Basahel^18^ examined the causal effects of leadership and attitudes on safety compliance and participation mediated by motivation and knowledge. Moshood et al.^19^ explained that construction projects often face numerous uncertainties that pose risks, such as time overruns, cost overruns, and poor quality delivery in building and road construction projects. Latif et al.^20^ studied the performance of TBMs by using digital twin and machine learning algorithms to minimize the risk associated with the cost and schedule of a tunneling project by simultaneously visualizing and monitoring. Long Li et al.^21^ studied the prediction of TBM cutter head speed and penetration rate for high-efficiency excavation of hard rock tunnels using a CNN-LSTM model with construction big data. Claudia Garrido Martins et al.^22^ studied the perceptions of construction risks due to fast-track activity overlapping. Zhang et al.^23^ studied the application of BIM technology in the risk management of tunnel project schedules. Li et al.^24^ used the monte carlo simulation method and uncertainty analysis method to study the uncertain risk of urban sewage pipe network. However, studies on the analysis and prediction of schedule risk in terms of refinement are presently limited, and the internal correlation of the prediction results and completion probability using the attitudes of the evaluators requires further investigation.

To the best of our knowledge, there are no existing studies on schedule risk analysis based on mood index prediction models, and refined research on schedule risks associated with underground engineering construction is lacking. Based on the uncertain factors affecting the construction period of the water intake tunnel, this study analyzes the influence of external risks and proposes a method to predict the expected construction period. Based on the attitude preference of the evaluator, the mood index is used to describe the emotion of the evaluator toward the project progress, and the construction period prediction model related to the mood index is deduced. First, progress analysis theory based on the PERT is introduced, and the shortcomings of the theory are outlined. Secondly, the risk model of the water intake tunnel schedule considering emotion factors is deduced, and the corresponding relationship between mood index and expectation probability, emotion grade, and expectation degree is established. Moreover, a construction period prediction model considering the types of surrounding rock is also constructed. Finally, engineering applications and analysis are conducted based on the least-squares principle, the relationship between pessimism, optimism, expected construction period, and completion probability is fitted respectively, and the model equation of the expected construction period and completion probability is obtained.

This study established an analysis model considering optimism and pessimism as evaluation factors to predict the expected water intake tunnel construction period and completion probability, thereby providing reference information to enable decision-makers to evaluate the risks and formulate mitigation measures.

## Schedule risk analysis theory

Tunnel construction schedule risk analysis evaluates the possibility of completion within a specified construction period or the risk of delay. Commonly used progress risk assessment methods include the critical path method and program evaluation and review technique (PERT). In particular, PERT can be used for the risk assessment of tunnel construction progress. The absolute difference between optimistic and pessimistic times in PERT can show the attitude of the tunnel project managers toward risk^15^. In a PERT schedule, each process has a duration $\beta$:

1 $${{\mu }_{j}=(a}_{j}+4{m}_{j}+{b}_{j})/6$$

2 $${\sigma }_{j}^{2}={({b}_{j}-{a}_{j})}^{2}/36$$

where ${a}_{j}$ is the optimistic time for the completion of process $(j)$ (in days (d)), ${m}_{j}$ is the most probable time for the completion of process $(j)$ (normal time) (in d), and ${b}_{j}$ is the pessimistic time for the completion of process $(j)$ (in d).

The duration ${T}_{n}$ and variance ${\sigma }_{n}^{2}$ of the critical path can then be obtained as:

3 $${T}_{n}=\sum_{j=1}^{n}{\mu }_{j}$$

4 $${\sigma }_{n}^{2}=\sum_{j=1}^{n} {\sigma }_{j}^{2}$$

where ${\sigma }_{n}$ is the mean-square deviation of the critical path.

The completion probability $P$ (in %) is subsequently determined using the following equation:

5 $$P=P(t\le {T}_{s})=\underset{-\infty }{\overset{{T}_{s}}{\int }}\frac{1}{{\sigma }_{n}\sqrt{2\pi }}exp(-\frac{1}{2}{\left(\frac{t-{T}_{n}}{{\sigma }_{n}}\right)}^{2}$$

where ${T}_{s}$ is the planned construction period (in d).

In the PERT progress analysis theory, the process evaluation is primarily based on experience. First, experts including the project managers, engineering technicians, and other professionals, estimate the construction period. The expected value and variance of the construction period are obtained from these estimates, and the completion probability is obtained using Eq. (5)^25^. In this method, the evaluators do not list the scoring basis in detail, and there is some uncertainty in the evaluation results. Hence, a detailed analysis of the attitudes and mood tendencies of the evaluator must be conducted to predict the risk using quantitative evaluation indicators.

## Schedule risk model of intake tunnel construction considering mood factors

### Analysis of factors influencing the construction period

For the water intake tunnel of a coastal power station, the factors that determine the construction period primarily include two aspects. First, manpower, resources, and equipment, and the adopted construction technology and process organization, determine the footage of each cycle which is relatively easy to calculate and estimate. These factors are direct decision factors that determine the absolute construction period of the project. Further, the changes and uncertain factors of the external environment, including factors at both subjective and objective levels, have a less deterministic effect on the schedule. The subjective level includes cross-operation and interaction with other surrounding units. The objective level includes changes in the geological conditions, blasting technology, and explosive supply, which are relatively more difficult to predict and evaluate than the direct decision factors.

### Preliminary prediction and analysis of expected construction period

The construction period for coastal power station projects is remarkably long, the subtasks and processes are closely related, and the risk of progress delay is high. Since the problems encountered during project construction share certain commonalities, data from completed projects can provide reference information for future projects. A tunnel project exhibits the following characteristics: a staggered layout and cross-construction in plane and space, numerous operating facilities around the tunnel and foundation pit, high requirements of the logical construction sequence, and a difficult overall project planning process^26^. Hence, progress control must be applied and a completed tunnel that is similar to the tunnel to be constructed must be evaluated.

The absolute construction period of the project can be evaluated based on the daily cyclic footage data obtained from the completed tunnel, which describe activities related to excavation, lining construction, and anticorrosion system installation, combined with the total length of the tunnel to be constructed and key processes. The differences in the absolute construction period indices determined by different evaluators are relatively small. Hence, this study primarily analyzed the external risks that affected the project.

For the convenience of the analysis, the completed reference unit and unit to be constructed are referred to as tunnels Nos. 1 and 2, respectively, in this study. First, the critical path of the water intake tunnel construction process was analyzed, and the factors affecting the construction progress were classified and identified using the structural decomposition method. The critical path of the water intake tunnel construction includes six steps: construction preparation → entrance into the tunnel → excavation and support → lining construction → grouting in the tunnel → tunnel wall repair and anticorrosion system installation (repair and anti-corrosion). The uncertain factors affecting progress are divided into the nine categories detailed in Table 1. This study analyzed the downtime (in d) caused by these nine factors.

**Table 1:** Uncertain factors affecting the progress of the water intake tunnel construction.

Item	Specific factors	Item	Specific factors
① Collapse, slippage	Collapse and slippage at the tunnel entrance or in the tunnel	⑥ Construction channel	Channel interruption caused by other surrounding projects
② Rain, snow	Construction transportation interruption caused by rainfall and snowfall	⑦ Cross-coordination	Small distance blasting crossing (blasting for other subtasks nearby and blasting of adjacent tunnels)
③ Technology, safety	Surrounding important subtasks of concrete pouring and concrete curing that limit blasting	⑧ Site handover	Delayed handover on connecting project site
④ Policy factors	Holidays and shutdown of explosives supply	⑨ Other	Welcoming inspection
⑤ Concrete supply	Delayed or limited concrete supply		

Assuming that there are $m$ types of uncertain factors affecting the tunnel construction period and $n$ items in the key construction process, in this study $m$=9 and $n$=6. Here, the probability of stoppage of the $j$ th process caused by the $i$ th factor of tunnel No. 1 is ${p}_{1ij}$, the overall probability of stoppage of the $j$ th process is ${p}_{1j}$, the absolute construction period for the $j$ th process is ${t}_{1j}$, and the downtime in d for the $j$ th process is ${d}_{1j}$. According to engineering experience, the possibility of the simultaneous occurrence of the different factors is quite small. Thus, an approximate expression for the stoppage probability can be formulated as expressed below:

6 $${p}_{1j}=\sum_{i=1}^{m} {p}_{1ij}$$

7 $${d}_{1j}={p}_{1j}{\cdot t}_{1j}$$

Assuming that the total absolute construction period for tunnel No. 1 is ${T}_{11}$ and the total downtime (in d) is ${T}_{12}$, the total construction period for tunnel No. 1 ${T}_{1}$ can be expressed as:

8 $${T}_{1}={T}_{11}+{T}_{12}=\sum_{j=1}^{n} {t}_{1j}+\sum_{j=1}^{n} {d}_{1j}$$

Denoting the absolute construction period of the $j$ th process for tunnel No. 2 as ${t}_{2j}$, the downtime (in d) of the $j$ th process as ${d}_{2j}$, the total absolute construction period as ${T}_{21}$, the total downtime (in d) as ${T}_{22}$, and the total construction period as ${T}_{2}$, if the $i$ th factor for tunnel No. 2 causes the probability of stoppage of the $j$ th process ${p}_{2j}$ to be similar to that of tunnel No. 1, the overall stoppage probability of the $j$ th process ${p}_{2j}$ can be expressed as follows:

9 $${p}_{2j}={p}_{1ij}$$

10 $${p}_{2j}=\sum_{i=1}^{m} {p}_{2ij}$$

11 $${d}_{2j}={p}_{2j}{\cdot t}_{2j}$$

12 $${T}_{2}={T}_{21}+{T}_{22}=\sum_{j=1}^{n} {t}_{2j}+\sum_{j=1}^{n} {d}_{2j}$$

### Construction period prediction model considering mood factors

Quiggin^27^ proposed the Rank-dependent Expected Utility (RDEU) theory that introduced the mood of players into the estimation process^28^. In the RDEU theory, the mood function is expressed as follows:

13 $$\omega \left({p}_{i}\right)={p}_{i}^{r}$$

where ${p}_{i}$ is the true probability of the event ${x}_{i}\mathrm{ occurring}$, ${p}_{i}\in$[0, 1], and $r$ the mood index.

When Eq. (13) is applied, the value of the mood index is related to the nature of the event of the research object. Herein, the nature of the event is divided into favorable and adverse events, wherein favorable events refer to events that are favorable for the realization of the goals and adverse events refer to those that are unfavorable for the realization of the goals.

When discussing favorable events, 0 < $r$  < 1 is used to describe the optimism of the decision-makers in Eq. (13). Thus, optimism would give favorable events a greater probability of occurrence. When $r$ > 1 for favorable events, the mood of the decision maker is considered to be pessimistic. Thus, pessimism can reduce the probability of occurrence of the favorable events. When $r$=1, the decision maker does not have any mood.

**Figure 1:**
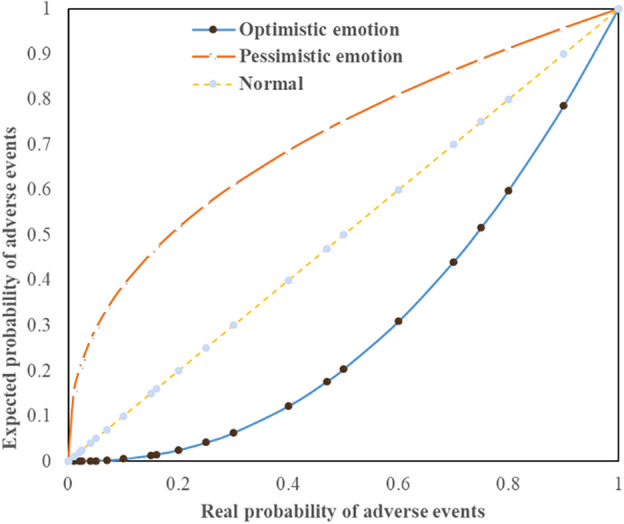
Mood function of the decision maker.

In the risk analysis of tunnel construction conducted in this study, the stoppage of the $j$ th process owing to the $i$ th factor is considered to be an adverse event. When encountering an adverse event, ${r}_{i}$ > 1, 0 < ${r}_{i}$  < 1, and ${r}_{i}$= 1 can be used to describe the optimism, pessimism, and lack of mood of the evaluator regarding the adverse event, respectively. Hence, when ${r}_{i}$ > 1, the evaluator is optimistic about the occurrence of the adverse event (they assign the adverse event a probability that is lower than that expected based on the neutral mood). When 0 < ${r}_{i}$  < 1, the evaluator is pessimistic about the occurrence of adverse events (they assign the adverse event a higher probability than that expected based on the neutral mood). When ${r}_{i}$= 1, the evaluator does not have any mood about the occurrence of the adverse events (they assign the probability expected based on the neutral mood). The relationship between the moods of the decision-makers and occurrence of adverse events is shown in Fig. 1, wherein the abscissa shows the real probability of the adverse event occurrence and the ordinate shows the expected probability of the adverse event occurrence, as determined based on the considerations of the influence of the mood. The mood index $r$ can be used to depict conveniently the emotional preference of risk managers and to quantify the numerous adverse factors that may affect the normal development of the project to provide a theoretical basis for the establishment of a fine quantitative analysis model of the expected construction period and completion probability.

The prediction and analysis of the expected construction period ${T}_{2}$ presented in this study was based on the evaluation of the construction period without considering the mood factors. When the attitude of the evaluators is considered during actual construction, the results will be remarkably different. Previous researchers divided evaluators into risk-taking^29,30^, neutral, and conservative types (also called risk tendency, risk-neutral, and risk aversion types, respectively). These three attitudes can be introduced into the project and the corresponding construction periods are then estimated as optimistic, normal, and pessimistic times accordingly.

To facilitate the analysis, the parameters $\alpha$ and $\beta$ (which represent the pessimism and optimism of the evaluator, respectively, as regards the occurrence of the events) were introduced into the risk analysis of the tunnel construction period. These parameters are collectively referred to as mood expectations, and their domain is defined as [0 100]. A higher $\alpha$ corresponds to a lower probability of favorable events and a higher probability of adverse events. Conversely, a higher $\beta$ corresponds to a greater probability of favorable events and a lower probability of adverse events.

The relationship between the mood index , expected probability, mood level, and expectation degree must be established to facilitate the quantitative analysis using the mood index. The expected probability corresponding to an original (real) probability of 0.5 was divided into eight intervals, wherein the dividing points matched the different mood grades and expectations. The corresponding mood index values represent a unified index for schedule risk analysis, and a comparison of the corresponding mood parameters is presented in Table 2.

**Table 2:** Comparison of mood parameters.

Mood state	Mood index $r$	Expected probability of the adverse event	Mood hierarchy	Expectation degree (symbol)
Pessimism	→ 0	1	Very pessimistic	100 (${\alpha }_{100}$)
	0.19	0.875	Pessimistic	75 (${\alpha }_{75}$)
	0.41	0.75	Somewhat pessimistic	50 (${\alpha }_{50}$)
	0.68	0.625	Slightly pessimistic	25 (${\alpha }_{25}$)
Normal	1	0.5	No mood	0 (${\alpha }_{0} or {\beta }_{0}$)
Optimism	1.4	0.375	Slightly optimistic	25 (${\beta }_{25}$)
	2	0.25	Somewhat optimistic	50 (${\beta }_{50}$)
	3	0.125	Optimistic	75 (${\beta }_{75}$)
	+ ∞	0	Very optimistic	100 (${\beta }_{100}$)

As shown in Table 2, the degrees of pessimism (and optimism) from no mood to very pessimistic (and very optimistic) were divided into four levels: the probabilities of the adverse effects corresponding to the pessimism degree values of 100, 75, 50, and 25 were defined as 1, 0.875, 0.75, and 0.625, respectively, thereby corresponding to the mood levels of the very pessimistic, pessimistic, somewhat pessimistic, and slightly pessimistic, respectively.

After considering the mood states of the decision-makers, the probability ${p}{^\prime}_{2ij}$ of the $i$ th factor causing the stoppage of process $j$ for the No. 2 tunnel is expressed as:

14 $${p}^{\prime}_{2ij}={p}_{2ij}^{r}$$

By combining Eqs. (9), (10), (11), (12), and (14), we obtain:

15 $${p}^{\prime}_{2j}=\sum_{i=1}^{m} {p}_{1ij}^{r}$$

16 $${T}_{2}=\sum_{j=1}^{n} {t}_{2j}+\sum_{j=1}^{n} {p}_{2j}^{\prime} \cdot {t}_{2j}$$

where ${p}_{2j}{\prime}$ is the probability of stoppage of the $j$ th process for tunnel No. 2 after considering the mood state of the decision-makers.

### Construction period prediction model considering surrounding rock types

Different problems encountered during the tunnel construction in terms of the tunnel length and burial depth arise from the geological uncertainty. Indeed, among the multiple factors that affect the progress and cost of tunnel construction, geological changes have the largest impact^2^. The method used to analyze the impacts of the different influencing factors on the stoppage of the tunnel construction was presented above. In the actual prediction of the construction period, the impact of the different risk preferences on the slowdown or acceleration of the progress must also be considered owing to the differences in the evaluation results caused by different assessments of the geological conditions.

The progress of the excavation (and support) process depends strongly on the type of surrounding rock. Although the geological survey report describes the distribution of the rock surrounding the tunnel axis, it cannot fully reflect the real situation owing to the limited number, density, and spacing of the boreholes. For example, during the excavation of a particular water intake tunnel, the author encountered a deep and strongly weathered fracture zone more than 10 m earlier than indicated by the geological survey report, thereby considerably affecting the progress of the project.

The boreholes used to develop the geological survey report of the tunnels are generally spaced in the range of 20–50 m. Thus, features of the surrounding rock that are less than 20 m long can be regarded as risk factors that may potentially affect the project progress. In the analysis of the degree of pessimism and optimism, the concern over the quality of the surrounding rock may increase or decrease based on the different risk preferences of the evaluators. According to the Code for Geotechnical Engineering Investigation (China), the surrounding rock can be divided into five classes (Classes I, II, III, IV, and V) from hard to soft (or from complete to broken), based the hardness and integrity of the rock. Assuming that the surrounding rock of a certain section is Class $N$ ($I \le N \le V$) and its length is less than 20 m, the surrounding rock class can be adjusted based on the risk preference, thereby limiting the maximum fluctuation of the rock class to within three grades. Thus, if the adjusted surrounding rock is class $M$, the following equations must be satisfied: $N-3 \le M \le N+3$ and $I \le M \le V$. No adjustments are made when $L \ge$ 20 m. The values of the surrounding rock classes subject to different expectations are presented in Table 3.

**Table 3:** Adjusted values of the surrounding rock classes based on expectations.

Item	Expectation	Expected surrounding rock class				
		When *N* = I	When *N* = *II*	When *N* = *III*	When *N* = *IV*	When *N* = *V*
$\alpha$	100	IV	V	V	V	V
	75	IV	V	V	V	V
	50	III	IV	V	V	V
	25	II	III	IV	V	V
$\beta$	100	I	I	I	I	II
	75	I	I	I	I	II
	50	I	I	II	II	III
	25	I	II	III	III	IV

Assuming a certain section of the *N*-class surrounding rock with length ${L}_{k}$ (${L}_{k} <$ 20 m) and daily excavation footage ${L}_{N}$, the surrounding rock class adjusted based on the expected attitude was denoted using the symbol $M$ and the daily excavation footage using the symbol ${L}_{M}$. Hence, the adjusted construction period deviation ${t}_{k}$ can be expressed as:

17 $${{t}_{k}=L}_{k}/{L}_{M}-{L}_{k}/{L}_{N}$$

When ${t}_{k}>$ 0, the quality of the surrounding rock is expected to decrease, thereby slowing down the progress. When ${t}_{k}<$ 0, the quality of the surrounding rock is expected to increase, thereby accelerating the progress. When ${t}_{k}=$ 0, the expected quality of the surrounding rock remains unchanged, thereby making the progress to remain normal. The construction period *t* affected by the change in the expected surrounding rock class is expressed as:

18 $${T}_{23}=\sum_{k=1}^{s}{t}_{k}$$

where $S$ is the number of surrounding rock sections with a length $L<$ 20 m.

By combining Eqs. (15), (16), and (18), the total construction period $T$ after considering the surrounding rock classes can be expressed as:

19 $${T=T}_{2}+{T}_{23}=\sum_{j=1}^{n} {t}_{2j}+\sum_{i=1}^{m}\sum_{j=1}^{n} {p}_{1ij}^{{r}_{i}} \cdot {t}_{2j} +\sum_{k=1}^{s}{t}_{k}$$

## Engineering application and analysis

To apply the model listed above in the actual project, the specific implementation idea is as follows. For a typical water intake tunnel project, firstly, the absolute construction period of each process of tunnel No. 2 can be obtained according to the measured daily average footage of each process of tunnel No. 1; secondly, after the statistical analysis of the factors causing the stoppage of tunnel No. 1, the probability of the stoppage caused by different factors can be obtained. Finally, the expected construction period and completion probability of tunnel No. 2 under different pessimism and optimism conditions are obtained by using the progress risk analysis theory of the emotional model and PERT.

### Project overview

The coastal water intake tunnel construction project considered in this study primarily provides seawater for cooling a conventional power plant. Units No. 1 and 2 of this power station each have one intake tunnel, with unit No. 1 placed into operation first. The lengths of the water intake tunnels for units No. 1 and 2 are 384 and 504 m long, respectively. Each tunnel is circular with an inner diameter of 6500 mm. Based on the daily average footage for each process measured during the construction of tunnel No. 1 (without external influence), the absolute construction periods for each process during the construction of tunnel No. 2 were determined and are presented in Table 4. The tunnel construction process begins with construction at one end, stops the excavation to install the lining once the excavation reaches certain footage, simultaneously begins excavation at the other end, and installs the lining from that end once the excavation is completed. The corresponding critical path and absolute construction period forecast are presented in Table 5. The stoppage probability of each process owing to different factors encountered during the construction of tunnel No. 2 can be obtained based on the statistical analysis of the stoppage factors encountered during the construction of tunnel No. 1, as presented in Table 6.

**Table 4:** Absolute construction period predictions for key processes of tunnel No. 2 construction.

Item	Excavation and support				Lining	Repair and anticorrosion
	Class II surrounding rock	Class III surrounding rock	Class IV surrounding rock	Class V surrounding rock		
Length (m)	394.00	18.00	30.00	48.00	92.00	92.00
Average daily footage (m)	4.00	2.50	1.00	0.60	3.15	5.00
Construction period (d)	98.50	7.20	30.00	80.00	29.21	18.40

**Table 5:** Absolute construction period forecast for each critical path process (d).

Process 1	Process 2	Process 3	Process 4	Process 5	Process 6
Construction preparation	Entrance into the tunnel	Excavation and support (inlet end and outlet end)	Lining	Grouting in the tunnel	Repair and anticorrosion
15	26	216	29	14	18

**Table 6:** Probability of the process stoppage based on the influencing factor.

Item	① Construction preparation	② Entrance into the tunnel	③ Excavation and support	④ Lining	⑤ Grouting in the tunnel	⑥ Repair and anticorrosion
① Collapse, slippage	0	0	0.067	0	0	0
② Rain, snow	0.05	0.038	0.038	0.038	0	0
③ Technology, safety	0	0.071	0.071	0	0	0
④ Policy factors	0	0.035	0.035	0	0	0
⑤ Concrete supply	0	0	0	0.164	0	0
⑥ Construction channel	0.012	0.012	0.012	0.012	0.012	0.012
⑦ Crosscoordination	0	0	0.022	0	0	0
⑧ Site handover	0.133	0	0	0	0	0
⑨ Other	0	0.01	0.01	0.01	0.01	0.01
Total	0.195	0.166	0.255	0.224	0.022	0.022

## Calculation results and analysis

**Figure 2:**
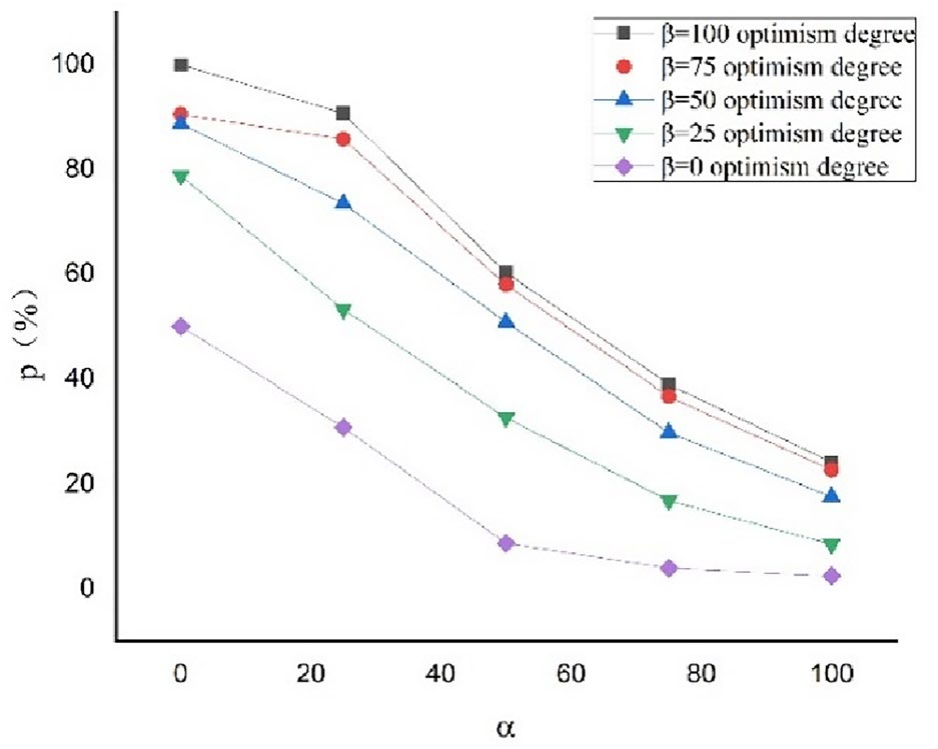
Relationships between the completion probability and degree of pessimism $\alpha$ at different degrees of optimism $\beta$.

**Figure 3:**
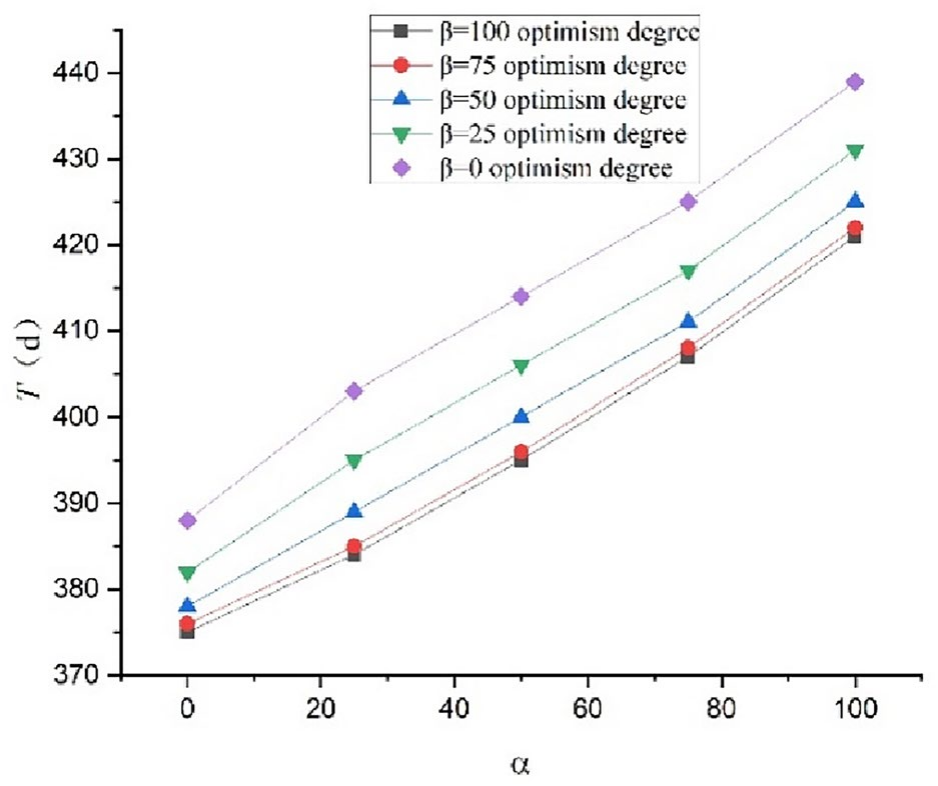
Relationships between the expected construction period and degree of pessimism $\alpha$ at different degrees of optimism $\beta$.

**Figure 4:**
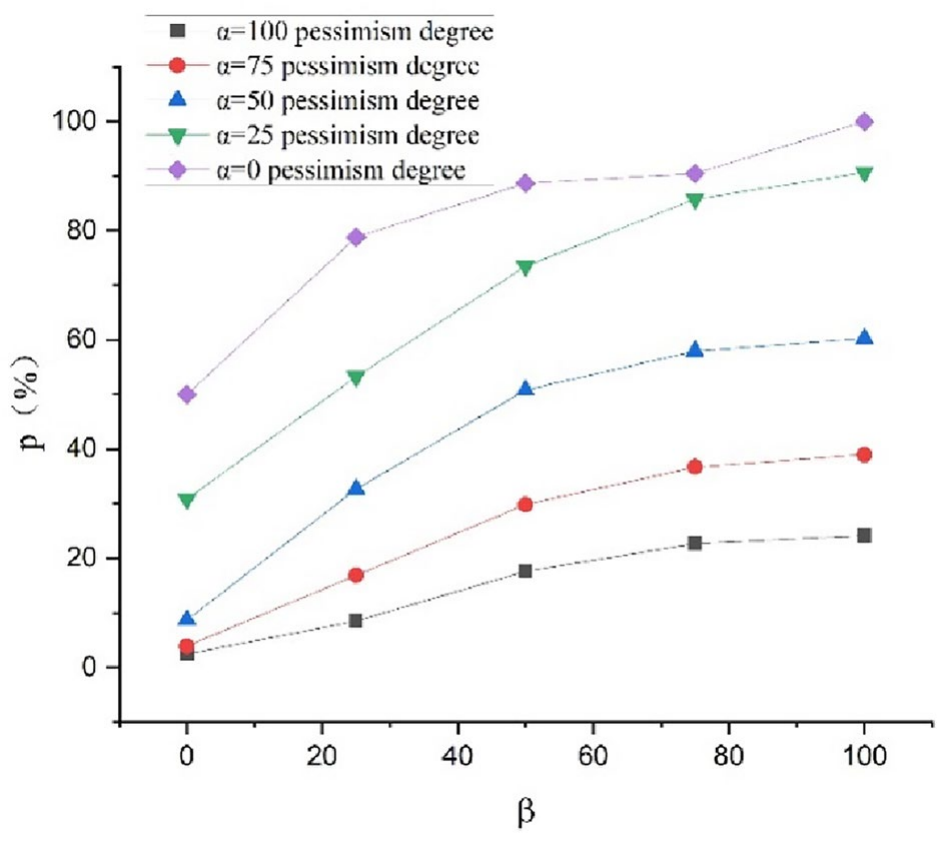
Relationships between the completion probability and degree of optimism $\beta$ at different degrees of pessimism $\alpha$.

**Figure 5:**
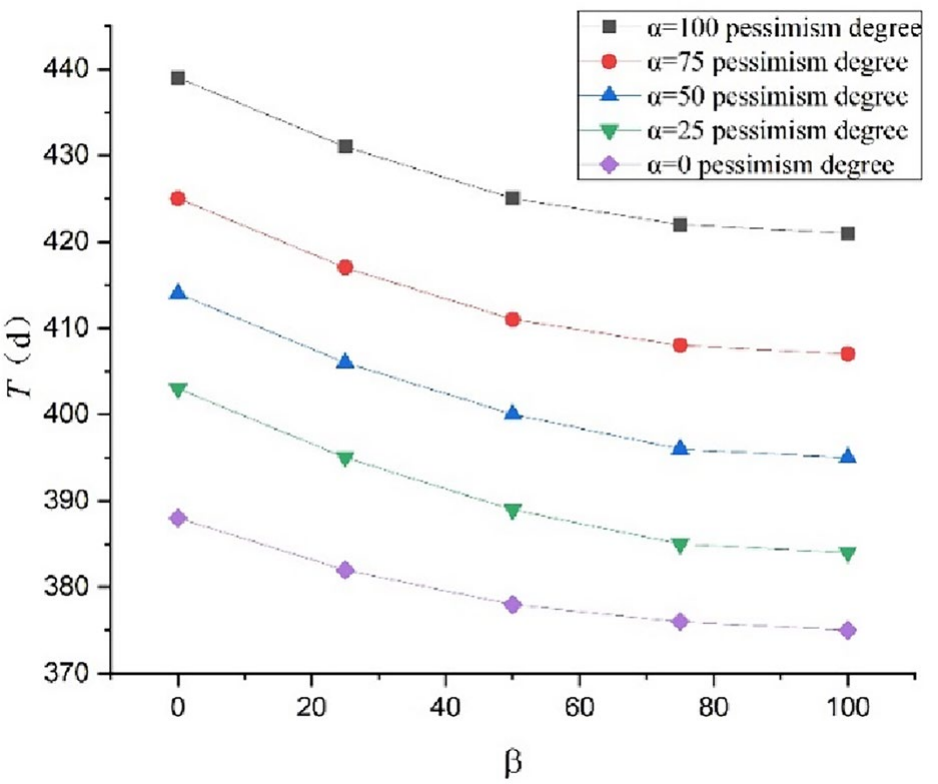
Relationships between the expected construction period and degree of optimism $\beta$ at different degrees of pessimism $\alpha$.

Using the schedule risk analysis theory with the proposed mood model combined with the PERT (Eqs. (1–5) in this study), the expected construction period subject to the combination of $\alpha$=100, 75, 50, 25, and 0 and $\beta$=100, 75, 50, 25, and 0 can be obtained. The outcomes are presented in Table 7. The completion probability can also be obtained for the planned construction period of ${T}_{s}$=400 d, as presented in Table 8. The curves expressing the relationship between the expected construction period and completion probability considering the pessimism for the different degrees of optimism are shown in Figs. 2 and 3, respectively. The curves expressing the relationship between the expected construction period and completion probability considering the optimism for the different degrees of pessimism are shown in Figs. 4 and 5, respectively. Figs. 2–5 show that the expected construction period increases and the probability of the project completion decreases with an increase in pessimism. Here, the opposite trend occurs with an increase in optimism.

**Table 7:** Expected construction period based on $\alpha$ and $\beta$.

Item index		Expected construction period (d)				
		$\beta$ condition				
		100	75	50	25	0
$\alpha$ condition	100	421	422	425	431	439
	75	407	408	411	417	425
	50	395	396	400	406	414
	25	384	385	389	395	403
	0	375	376	378	382	388

**Table 8:** Completion probability based on $\alpha$ and $\beta$.

Item index		Completion probability (%)				
		$\beta$ condition				
		100	75	50	25	0
$\alpha$ condition	100	24.20	22.66	17.62	8.51	2.44
	75	38.98	36.70	29.81	16.86	3.93
	50	60.26	57.93	50.80	32.64	8.70
	25	90.66	85.77	73.48	53.25	30.86
	0	99.98	90.49	88.69	78.81	50.00

**Figure 6:**
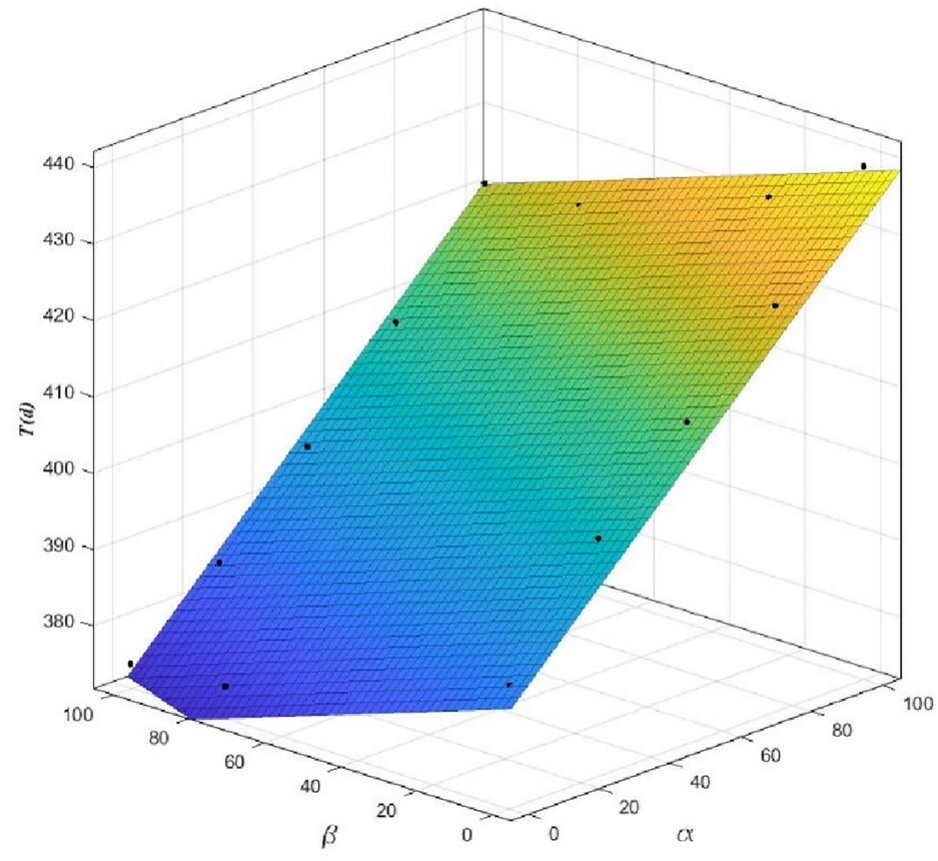
Fitting surface of the expected construction period *T* as functions of $\alpha$ and $\beta$.

**Figure 7:**
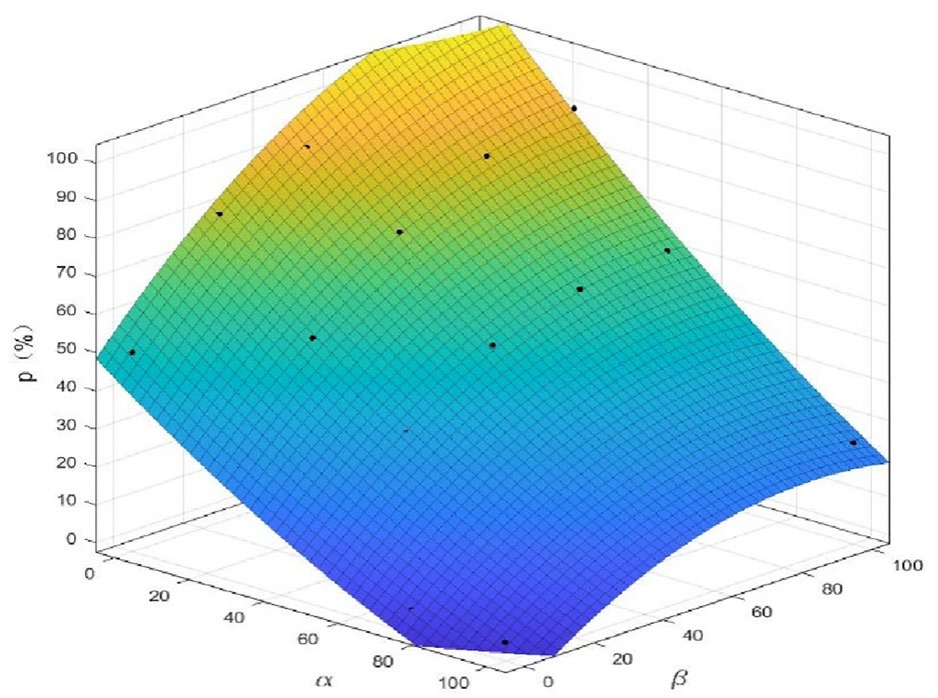
Fitting surface of the completion probability* p* as functions of $\alpha$ and $\beta$.

To determine the change in the expected construction period and completion probability with the attitude of the evaluators more precisely, the relationships between $\alpha$ and $\beta$ and the expected construction period and completion probability were fitted based on the least-squares principle, and the corresponding equations were obtained, as presented in Table 9. When the expected construction period was expressed by a linear function of $\alpha$ and $\beta$, the fitting degree reached 0.9848. The fitting surface is shown in Fig. 6. When the expected construction period was expressed by a quadratic function of $\alpha$ and $\beta$, the fitting degree reached 0.9754. The fitting surface shown in Fig. 7. Thus, when predicting the construction period and probability of completion, the evaluators can accurately quantify the evaluation process and results using the fitting equations in Table 9. For example, when the evaluator expresses the mood values of $\alpha$=75 and $\beta$=90, the expected construction period and completion probability are 408 d and 40.64%, respectively. Thus, the proposed method provides more accurate reference information, thereby allowing the decision-makers to evaluate the risks and establish a scientific basis for the formulation of the mitigation measures.

**Table 9:** Fitting results for the expected construction period and probability of completion.

Fitting method	Expected construction period$T$(d)	${R}^{2}$
Linear fitting ($\alpha$and$\beta$are both first power)	$T=388+0.472 \alpha -0.1744\beta$	0.9848
Completion probability$P$(%)		
Linear fitting ($\alpha$and$\beta$are both first power)	$P=59.72-0.6983\alpha +0.4294\beta$	0.9361
Nonlinear fitting ($\alpha$is first power and$\beta$is second power)	$P=47.56-0.5622\alpha +0.9939\beta -0.002722\alpha \cdot \beta -0.004283{\beta }^{2}$	0.9714
Nonlinear fitting ($\alpha$is second power and$\beta$is first power)	$P=55.2-0.7447\alpha -0.5655\beta -0.002722\alpha \cdot \beta +0.001825{\alpha }^{2}$	0.9531
Nonlinear fitting ($\alpha$and$\beta$are both second power)	$P=49.84-0.7447\alpha +0.9939\beta +0.001825{\alpha }^{2}-0.002722\alpha \cdot \beta -0.004283{\beta }^{2}$	0.9754

## Conclusion

This study investigated the incorporation of a mood index in the prediction of the construction period and the probability of completion for the water intake tunnels. The following key conclusions were obtained:

- (1) For the prediction of risk to the water intake tunnel construction schedules, the mood index can be used to describe the perception of the evaluator on the progress of the project using three states (pessimism, optimism, and no mood). Here, a model related to this mood index can be used to predict the expected construction period. Definitions of the degrees of pessimism $\alpha$ and optimism $\beta$ regarding the occurrence of events were introduced and were collectively referred to as mood expectation.
- (2) The expected construction period increased and the completion probability decreased considerably with an increase in pessimism. Here, the opposite trend was observed with an increase in optimism.
- (3) The expected construction period and completion probability can be expressed using the prediction model using $\alpha$ and $\beta$. When the expected construction period was expressed by a linear function of $\alpha$ and $\beta$, the fitting degree was 0.98. When the completion probability was expressed as a quadratic function of $\alpha$ and $\beta$, the fitting degree was 0.97.
- (4) The prediction model can be used during the process of risk control to conduct a detailed quantitative analysis of the expected construction period and completion probability, thereby providing accurate reference information that allows the decision-makers to evaluate the project risks effectively.

## Data Availability

All data generated or analyzed during this study are included in this published article.
